# Characterization of weaning-induced breast involution in women: implications for young women’s breast cancer

**DOI:** 10.1038/s41523-020-00196-3

**Published:** 2020-10-16

**Authors:** Sonali Jindal, Jayasri Narasimhan, Virginia F. Borges, Pepper Schedin

**Affiliations:** 1 Department of Cell, Developmental and Cancer Biology, Oregon Health & Science University, 2720 S Moody Avenue, Mailing Code: KR-CDCB, Portland, OR 97201 USA; 2 Knight Cancer Institute, Oregon Health & Science University, 2720 S Moody Avenue, Mailing Code: KR-ADM, Portland, OR 97201 USA; 3 Division of Medical Oncology, School of Medicine, University of Colorado Anschutz Medical Campus, MS8117, RC-1S, 8401K, 12801 E 17th Avenue, Aurora, CO 80045 USA; 4 Division of Medical Oncology, Young Women’s Breast Cancer Translational Program, School of Medicine, University of Colorado Anschutz Medical Campus, MS8117, RC-1S, 8401K, 12801 E 17th Avenue, Aurora, CO 80045 USA

**Keywords:** Breast cancer, Cancer microenvironment, Cancer prevention, Developmental biology, Target identification

## Abstract

In rodents, weaning-induced mammary gland involution supports increased mammary tumor incidence, growth, and progression to metastasis. Further, the protumor attributes of gland involution are COX-2 dependent and mitigated by short-duration non-steroidal anti-inflammatory drugs (NSAIDs), suggesting a potential prevention strategy. However, the transition from lactation to postweaning breast involution has not been rigorously evaluated in healthy women. Here we queried breast biopsies from healthy women (*n* = 112) obtained at nulliparity, lactation, and multiple postweaning time points using quantitative immunohistochemistry. We found that mammary remodeling programs observed in rodents are mirrored in the human breast. Specifically, lactation associates with the expansion of large, secretory mammary lobules and weaning associates with lobule loss concurrent with epithelial cell death and stromal hallmarks of wound healing, including COX-2 upregulation. Altogether, our data demonstrate that weaning-induced breast involution occurs rapidly, concurrent with protumor-like attributes, and is a potential target for NSAID-based breast cancer prevention.

## Introduction

Breast cancer diagnosed within 10 years of a completed pregnancy, a period of increased breast cancer risk in women, is called postpartum breast cancer (PPBC)^1–4^. It is well recognized that young breast cancer patients, especially women who recently completed a pregnancy, are at high risk of developing metastatic disease^5–10^. Utilizing rodent models of PPBC, postpartum weaning-induced mammary gland involution has been identified as a potential driver of the increased incidence and poor prognosis of breast cancer observed in young parous women^3,11,12^. Weaning-induced involution is a programmed cell death and stromal remodeling process that returns the lactation-competent mammary gland to its pre-pregnant, non-lactational state^11,13^. In rodent models, histological evidence of weaning-induced mammary lobule loss occurs 1–3 days after synchronized weaning, with extracellular matrix remodeling and adipocyte repopulation occurring gradually, as early as 2 days after weaning^14–19^. During the involution process, programmed cell death eliminates ~80–90% of the secretory mammary epithelium within a tissue microenvironment skewed towards wound healing^11,13–15,20,21^. Specifically, studies show that postweaning mammary glands have increased lymphangiogenesis^11,22^, fibroblast activation^23,24^, fibrillar extracellular matrix deposition^25^, and enrichment for CD11b+/F480+ myeloid-derived suppressor cells (MDSCs), M2-skewed macrophages^21,26^, and Th-17, Th-2, and Treg skewed T-cells^27^ compared to nulliparous glands.

Studies in murine models show that the mammary microenvironment during weaning-induced involution supports increased tumor incidence and growth, as well as progression to metastatic disease^11,12,21,23,28,29^. Further, preclinical studies show that the pro-tumorigenic processes of weaning-induced involution are cyclooxygenase-2 (COX-2) dependent and mitigated by moderate dose, short-duration ibuprofen treatment targeted to the involution window^12,28,30^. Importantly, a recent study in non-tumor-bearing rodents showed that ibuprofen treatment during involution did not affect weaning-induced lobule regression, reduce the ability of treated dams to successfully raise pups from a subsequent pregnancy, result in autoimmunity, nor impact physiological T-cell suppression that occurs in the normal involuting gland^28^. Assuming weaning-induced breast remodeling occurs similarly in women, these rodent data support the potential efficacy of a non-steroidal anti-inflammatory drug (NSAID) based postweaning pill to mitigate the increased risk of breast cancer following a recent pregnancy.

Published literature regarding weaning-induced breast involution in women is disparate, with some studies reporting the persistence of large, differentiated lobules that expand during pregnancy until peri-menopause^31,32^ and other studies reporting dramatic loss of these lobules within 18 months after childbirth^11^. These studies have been limited by small sample size^31,32^, reliance on normal tissue adjacent to breast cancer as a surrogate for true-normal tissue^11^, and a lack of lactation history^11^. Here we used tissue collected from two prospective, non-intervention biopsy trials of healthy women between 20 and 45 years of age. The first set of tissue was from a study of weaning-induced breast involution in women (*n* = 64) who provided detailed lactation history and a single breast biopsy at a randomized time point of 0.5, 1, 2, 3, 4, or 6–12 months post-wean. The second set included breast biopsies of nulliparous, lactational, and >12 months post-wean women (*n* = 48) obtained from the Komen Tissue Bank. Using quantitative histological and immunohistological analyses, we investigated lobular composition, milk protein expression, epithelial cell death, CD45+ immune cell infiltrate, lymphangiogenesis, lymphatic function, and COX-2 expression. Altogether, our data demonstrate that, in women, lobules that expand during pregnancy to accommodate lactation are eliminated after weaning. In addition, our results show that this process occurs rapidly, within 3 months of weaning, and is characterized by a wound-healing-like microenvironment with COX-2 upregulation. These data support the hypothesis that weaning-induced involution is a transient window of increased breast cancer risk that might be mitigated by non-steroidal anti-inflammatory treatment targeted to the ~3-month post-wean window of active tissue remodeling.

## Results

### Lobular composition of the breast is reduced by 0.5 months post-wean and returns to a pre-pregnant-like state by 3 months post-wean

**Fig. 1:**
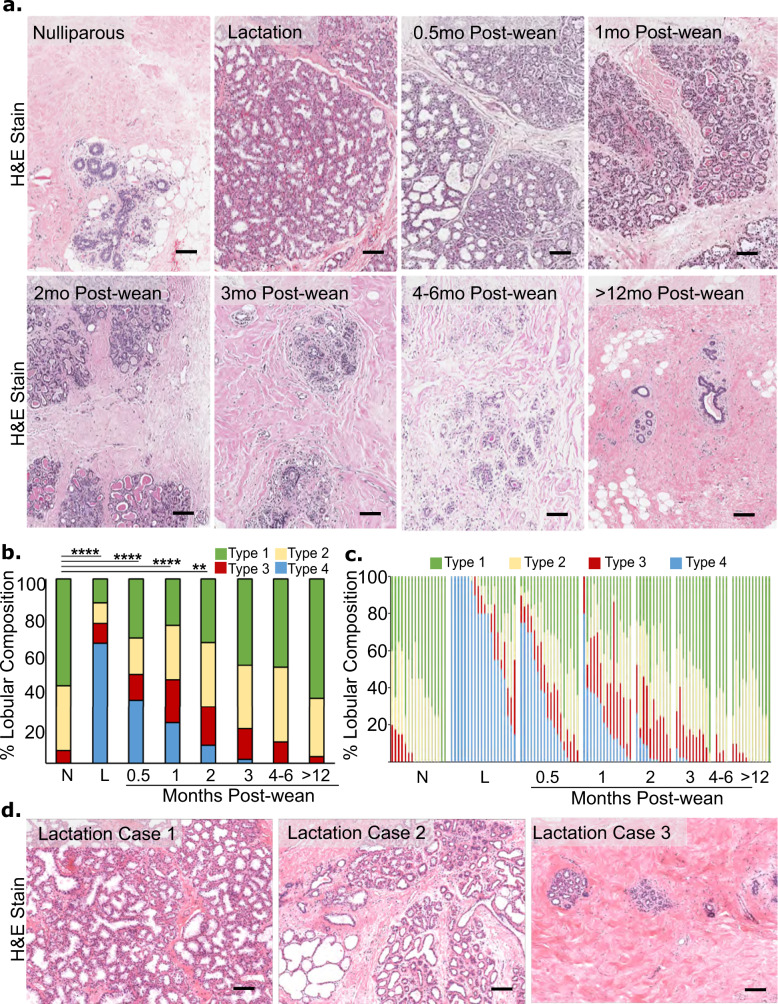
Breast lobular composition returns to pre-pregnant like state by three months post-wean. **a** Representative H&E images of biopsies from each reproductive group: nulliparous, lactation, and 0.5, 1, 2, 3, 4–6, >12 months post-wean. **b** Quantification of the average lobular composition by lobular subtype for each group: nulliparous (N) *n* = 17, lactation (L) *n* = 20, and 0.5 (*n* = 18), 1 (*n* = 15), 2 (*n* = 11), 3 (*n* = 11), 4–6 (*n* = 4), 12–24 months post-wean (*n* = 12). In this chort, type 1 average 15 acini/lobule, type 2 average 35 acini per lobule, type 3 average 112 acini/lobule, and type 4 average 112 acini/lobule with flattened epithelium and large acinar lumen. **c** Lobular **c**omposition by case shows heterogeneity within each reproductive group. **d** Representative H&E images show lobular heterogeneity observed in the lactation group. (****P ≤ 0.0001, **P ≤ 0.01, scale bar = 100 µm).

We first determined the time course of weaning-induced breast involution in women using quantitative morphological and immunohistochemical assessments of glandular composition and function. To assess lobular composition in this cohort, we performed morphometric analysis on each woman’s individual breast biopsy to identify how long post-wean it takes the breast to return to a nulliparous-like state at the cohort level. Lobular subtype was determined by pathological assessment based on number of acini and morphologic characteristics of the epithelium. Lobular subtypes were defined as type 1 (1–15 acini per lobule), type 2 (16–50 acini per lobule), type 3 (more than 50 acini per lobule), and type 4 (lobules with a secretory morphology), as previously described^11,31^. In this cohort, we find lobular composition to be highly dependent on reproductive categories and time since weaning (Fig. 1a). The nulliparous gland is characterized by small type 1 and type 2 lobules that lack secretory morphology^11^. In the lactating gland, the dominant lobule subtypes are large type 3 lobules and type 4 lobules with histological evidence of milk-filled lumen and characteristic flattened epithelia. Reduced type 4 and increased type 3 and 2 lobules compared to lactation were observed at the earliest postweaning time point analyzed (0.5 months), with further reduction in type 3 and 4 lobules at 1 and 2 months post-wean (Fig. 1a). By 3 months post-wean, breast composition was histologically similar to the nulliparous group (Fig. 1a). To quantify these histological observations, six cores from each case were assessed morphologically for total lobule number and type, resulting in the analysis of ~9000 lobules across the entire tissue cohort. This analysis confirmed that nulliparous tissue was mainly comprised of type 1 and 2 lobules, with some type 3 lobules and no type 4 lobules (Fig. 1b). During lactation, type 3 and 4 lobules make up to ~80% of the tissue analyzed (Fig. 1b), a 13-fold increase compared to nulliparous tissue. In postweaning breast tissue, we saw a sharp decrease in type 4 lobules compared to lactation, with ~50% fewer type 4 lobules at 0.5 months post-wean (Fig. 1b). We found that the lobular composition of postweaning breast tissue is statistically different from nulliparous tissue only up to 2 months post-wean (*P* < 0.0001, Fig. 1b). At 3 months post-wean and thereafter, the lobular composition of the breast is statistically indistinguishable from the nulliparous gland (Fig. 1b). Altogether, these quantitative morphology data support the idea that the secretory lobules expanded during pregnancy undergo full involution between 2 months and 3 months post-wean.

We next determined intra-group heterogeneity of lobular composition to better understand the natural variation found in women. In nulliparous women, the vast majority of lobules were types 1 and 2. However, ~40% of nulliparous cases had some type 3 lobules, with a few cases showing up to 20% type 3 lobules (Fig. 1c). As anticipated, type 4 lobules dominate the breast tissue in most lactation cases (Fig. 1b). However, even during lactation, lobular composition was variable, ranging from 100% type 4 lobules to <20% type 4 lobules. In fact, ~20% of actively lactating women had <60% combined type 3 and 4 lobules at time of biopsy, with the remaining lobules being type 1 and 2 (Fig. 1c). Representative hematoxylin and eosin (H&E) stained images show the variable breast lobule phenotypes observed in nursing women (Fig. 1d). One potential explanation for this inter-case heterogeneity during lactation is that lobular composition is influenced by length of total lactation and/or by length of exclusive nursing. However, statistical analysis of the lactation data revealed that total duration of lactation, duration of exclusive nursing, and number of previous pregnancies had no relationship with lobular composition at 0.5 months post-wean (Supplementary Fig. 1). These data support that the variation we observed in lobular composition among lactation cases is unrelated to lactation history.

### Adipophilin expression is elevated in all lobular subtypes during lactation and is sharply reduced at 0.5 months post-wean

**Fig. 2:**
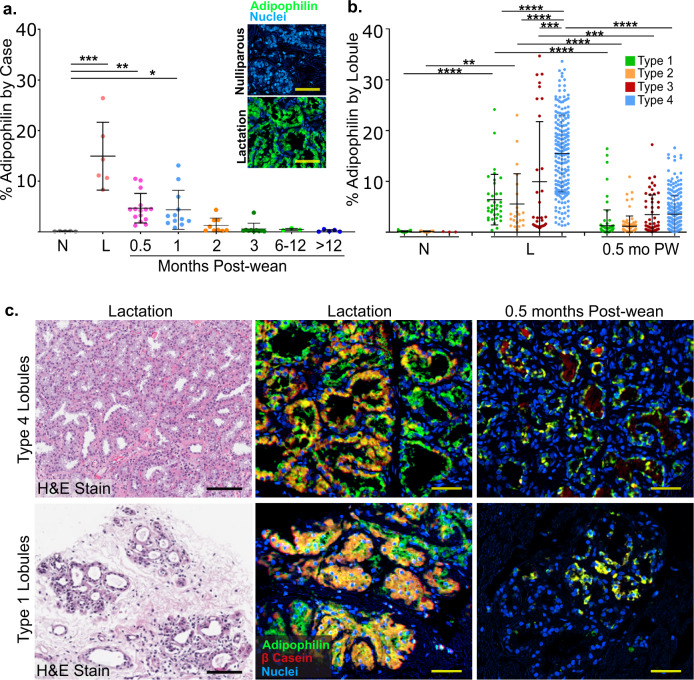
Expression of the milk proteins adipophilin and β-casein occurs in all lobular subtypes at lactation with rapid decline by 0.5 months post-wean. **a** Percent of adipophilin expression per mm^2^ for each group by case including nulliparous (N) *n* = 5, lactation (L) *n* = 6, and 0.5 (*n* = 15), 1 (*n* = 12), 2 (*n* = 9), 3 (*n* = 10), 6–12 (*n* = 3), 12–24 months post-wean (*n* = 5). Pseudo color multiplex immuno histochemistry (mIHC) images of adipophilin staining (green) in nulliparous (negative control) and lactational (positive control) glands are shown. **b** Percent of adipophilin expression per mm^2^ in nulliparous, lactation and 0.5 months post-wean cases by lobular subtype. **c** Representative H&E-stained images for type 4 and type 1 lobules in lactation cases (left column). mIHC stain for adipophilin (green) and β-casein (red) in type 4 and type 1 lobules during lactation (middle column) and at 0.5 months (right column) shows co-expression at the single cell level (orange/yellow). (****P ≤ 0.0001, ***P ≤ 0.001, **P ≤ 0.01, *P ≤ 0.05, scale bar = 50 µm).

Lobular composition of the breast is only one measure of lactation function and breast involution. In order to more fully answer the question of how long post-wean it takes for the breast to return to its pre-secretory, nulliparous-like state, we stained for adipophilin, a protein essential for milk lipid secretion^33^. We assessed adipophilin expression in representative cases across all groups (*n* = 65). As expected, we found adipophilin expression was near zero in the breast tissue of nulliparous cases and was highest during lactation (Fig. 2a). At 0.5 months post-wean, adipophilin expression was already reduced by 70% compared to lactation (Fig. 2a). Compared to nulliparous glands, adipophilin expression remained significantly elevated at 1 month post-wean, but returned to near nulliparous levels by 2 months post-wean (Fig. 2a, *P* < 0.02).

To assess what types of lobules produce milk, we quantified adipophilin expression by lobular subtype. During lactation, we found that type 4 lobules expressed the highest amount of adipophilin; however, even small type 1 and 2 lobules expressed adipophilin (Fig. 2b). To further explore the lactational proficiency of type 1 and 2 lobules, we dual-stained for adipophilin and β-casein, a second milk protein marker. During lactation, type 1 and 2 lobules (Fig. 2c, bottom row, middle panel) expressed both adipophilin (green) and β-casein (red) similar to type 4 lobules (Fig. 2c, top row, middle panel). Further, at 0.5 months post-wean, all lobule types had reduced staining for adipophilin and β-casein compared to lactation (Fig. 2c, right panels). Together, these data indicate that milk production can occur in lobules regardless of subtype, although type 4 lobules express the highest levels of adipophilin per unit area (Fig. 2b), suggesting increased lipid secretion proficiency.

### Hallmarks of a wound-healing microenvironment peak at 0.5 months post-wean

**Fig. 3:**
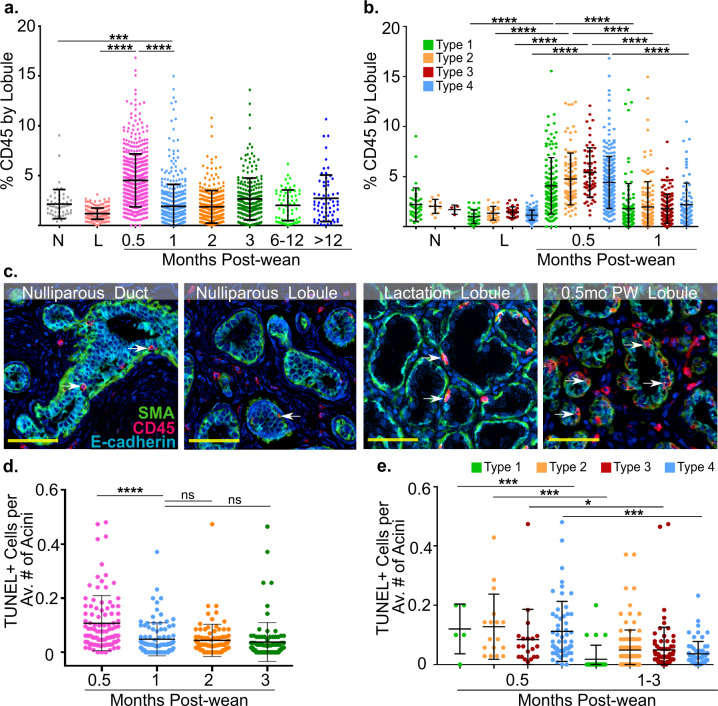
Hallmarks of postweaning involution occur in all lobular subtypes and peak at 0.5 months post-wean. **a** CD45+ expression by lobule for each group including nulliparous (N), *n* = 5, lactation (L) *n* = 5, and 0.5 (*n* = 15), 1 (*n* = 12), 2 (*n* = 9), 3 (*n* = 10), 6–12 (*n* = 3), 12–24 months post-wean (*n* = 5). **b** CD45+ expression by lobular subtype for nulliparous, lactation, and 0.5 months post-wean cases. **c** Representative pseudocolor multiplex IHC images of nulliparous (duct and lobule), lactation (lobule) and 0.5 months post-wean (lobule) showing spatial relationships between CD45+ immune cells (red), E-cadherin+ epithelial cells (blue), and SMA+ myoepithelium (green). Arrows show immune cells interspersed within acinar epithelium. **d** TUNEL+ epithelial cells per average number of acini are shown at 0.5 (*n* = 6), 1 (*n* = 6), 2 (*n* = 6), and 3 (*n* = 6) months post-wean. **e** Number of TUNEL+ epithelial cells per average number of acini at 0.5 and 1–3 months post-wean is shown by lobular subtype (****P ≤ 0.0001, ***P ≤ 0.001, *P ≤ 0.05, scale bar = 50 µm).

An identified hallmark of weaning-induced mammary gland involution in rodents is increased immune cell infiltrate, including cells of both myeloid and lymphoid lineages^13,26,28^. To determine whether breast tissue is inflamed during weaning-induced involution, and to identify the time course of inflammation, we stained our cohort for CD45, a transmembrane protein found on all differentiated immune cells^34^ that has been used to capture immune cell infiltration in previous studies^11,13^. We found moderate levels of CD45+ immune infiltrate in breast tissue from nulliparous women, which was reduced at lactation (Fig. 3a). At 0.5 months post-wean, we saw a significant ~3-fold increase in CD45 staining compared to lactation, consistent with breast involution being a developmentally regulated, inflammatory process (Fig. 3a, *P* < 0.001). CD45 expression decreased as early as 1 month post-wean (*P* < 0.001) and was not different from nulliparous tissue or later postweaning time points (Fig. 3a). These data suggest inflammation plays an early role in weaning-induced breast involution, but is largely resolved after 1 month post-wean (Fig. 3a). Next, we determined whether the CD45+ immune cell influx was confined to the large type 3 and 4 lobules or if all lobules become inflamed with weaning. We found elevated CD45+ staining in all lobular subtypes at 0.5 months compared to lactation (Fig. 3b, *P* < 0.01). These results indicate that inflammation occurs in all lobular subtypes and are consistent with our milk protein and lobular composition data, which found that the period between weaning and 1 month post-wean is the most active window of gland involution in all lobular subtypes.

As our data showed that CD45+ immune cells in the breast at 0.5 months post-wean were increased compared to nulliparous tissue, we assessed the spatial relationship between this immune population and the breast epithelium. A representative image of a nulliparous case showed that the majority of CD45+ cells are interspersed within the inter-acinar space, with a few CD45+ cells appearing in direct contact with the acinar and ductal epithelium (Fig. 3c). However, at 0.5 months post-wean, the number of CD45+ immune cells in close association with acinar epithelium was markedly increased (Fig. 3c). We quantified the ratio of intraepithelial to intralobular stromal CD45+ cells in nulliparous (*n* = 5) and 0.5 months post-wean (*n* = 5) cases. We found ~4-fold more intraepithelial to intralobular stromal CD45+ cells in 0.5 months post-wean cases. Specifically, we found an average of 2.17 intraepithelial CD45^+^ cells to every stromal CD45+ at 0.5 months compared to 0.51 intraepithelial CD45+ cells to every stromal CD45+ cell in nulliparous cases. This difference reached statistical significance (*p* < 0.016). During lactation, calling the location of CD45+ cells as either intraepithelial or intralobular stroma was difficult to due to epithelial density and sparse intralobular stroma (Fig. 3c). Therefore, to interrogate the location of CD45+ cells during lactation would require a future study with basement membrane staining. Together, these data suggest active immune function in the normal, healthy breast at all reproductive categories evaluated, with increased intraepithelial and stromal inflammation coinciding with active lobule loss occurring in the first month post-wean.

We next assessed the role of epithelial cell death in lobule loss by performing terminal deoxynucleotidyl transferase dUTP nick-end labeling (TUNEL) staining, which identifies double-stranded DNA breaks associated with apoptosis. We found high numbers of TUNEL+ epithelial cells at 0.5 months post-wean, with a sharp decline in TUNEL positivity at 1 month post-wean (Fig. 3d, *P* < 0.0001). Further, at 0.5 months, the number of TUNEL+ cells peaked in all lobular subtypes compared to 1–3 months (*P* < 0.04, Fig. 3e). These data show that two hallmarks of weaning-induced mammary gland involution in rodents, CD45+ immune cell infiltrate and TUNEL+ epithelial cell death, peak at 0.5 months post-wean in all lobular subtypes, even small type 1 lobules.

### Lymphatic density and function increase during early weeks of weaning-induced breast involution

**Fig. 4:**
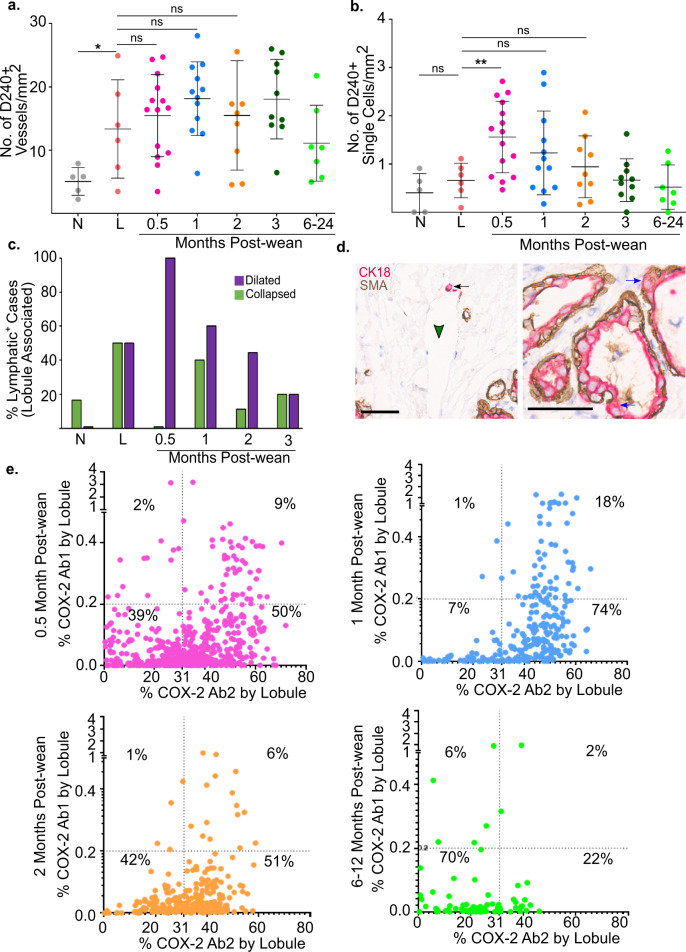
Lymphatic density and function increase during early weeks of weaning-induced involution. **a** Lymphatic density measured by D2–40+ vessels/mm^2^ for each group by case including nulliparous (N) *n* = 5, lactation (L) *n* = 6, and 0.5 (*n* = 14), 1 (*n* = 12), 2 (*n* = 9), 3 (*n* = 10), 6–24 months post-wean (*n* = 7). **b** Number of D2–40+ single cells/mm^2^ for nulliparous (N) *n* = 5, lactation (L) *n* = 6, and 0.5 (*n* = 15), 1 (*n* = 12), 2 (*n* = 9), 3 (*n* = 10), 6–24 months post-wean (*n* = 7) cases. **c** Percentage of cases with dilated (purple) and collapsed (green) lymphatics present within 100 um of lobules in nulliparous (N) *n* = 5, lactation (L) *n* = 6, and 0.5 (*n* = 6), 1 (*n* = 9), 2 (*n* = 9), 3 (*n* = 10), 12–24 (*n* = 6) months post-wean. **d** Breast biopsy at 0.5 months post-wean stained for CK18 (red), SMA (brown), and hematoxylin counterstain (blue) showing CK18+ signal within mammary lymphatics (left panel) and basally located mammary epithelial cells outside the confines of the SMA+ myoepithelial cell layer (right panel). **e** Dot plots of lobules with positive COX-2 expression using Ab1 and Ab2 for 0.5, 1, 2, and 6–12 months post-wean. The K-means high and low COX-2 cutoff value (Ab1 = 0.2%, Ab2 = 31%) are shown by the dotted line (***P ≤ 0.001, **P ≤ 0.01, *P ≤ 0.05, scale bar = 50 µm).

Involution-associated lymphatics have been implicated in the clearance of milk and dying cells in rodent mammary glands^11^. However, lymphangiogenesis has not been demonstrated in the normal postweaning human breast. Importantly, it is well established that increased lymphatic density correlates with metastatic risk in PPBC^11,22^, making the study of lymphangiogenesis in the normal breast of particular interest. To differentiate blood vessels from lymphatics, we stained for the lymphatic-specific endothelial cell surface marker, podoplanin (D2–40)^35^. We found increased lymphatic vessel density in the lactation group, suggesting increased capacity for fluid drainage compared to nulliparous tissue (Fig. 4a). Consistent with an active role for lymphatics during breast involution, the number of D2–40+ vessels/mm^2^ was highest in the 1 month post-wean group (Fig. 4a). However, lymphatic density alone is not sufficient to infer active lymphangiogenesis during involution. Since single D2–40+ lymphatic cells have been shown in preclinical models to precede new lymphatic formation^11^, we assessed the number of single cells that stained for D2–40. We found that single D2–40+ cells peaked at 0.5 months post-wean (Fig. 4b), prior to the peak in lymphatic density observed at 1 month post-wean (Fig. 4a). These data are consistent with new lymph vessel formation. At 1 month post-wean, single D2–40+ cells drop to levels not statistically different from lactation, as would be expected if the single D2–40+ cells we observe at 0.5 months post-wean were contributing to the increase in lymphatic vessels observed at 1 month post-wean. Interestingly, we did not see an increase in single D2–40+ lymphatic cells during lactation, suggesting that the processes mediating increased lymphatic density during lactation may occur earlier, during pregnancy (Fig. 4b).

To address lymphatic function, we utilized the classic assignment of vessel dilation (carrying fluid) or collapse (not carrying fluid). First, by pathologist’s assessment, ~80% of nulliparous cases did not have lobule-associated lymphatics (Fig. 4c). Of the 20% of nulliparous cases containing perilobular lymphatics, almost all of the lymphatics were defined as collapsed (Fig. 4c). During lactation, most cases had perilobular lymphatics, with a ~50% split between cases with lymphatics defined as collapsed or dilated (Fig. 4c). In cases at 0.5 months, we found all lymphatic vessels to be dilated (Fig. 4c). With increased time post-wean, the percentage of cases with dilated perilobular lymphatics decreased, returning to near nulliparous levels by 3 months post-wean (Fig. 4c). These data suggest that lymphatics play an active role during involution by providing increased tissue drainage, an observation further supported by the presence of cellular debris within the breast lymphatic vessels. At 0.5 months post-wean we found numerous examples of lymphatics (Fig. 4d, green arrowheads) containing CK18+ epithelial cell debris (Fig. 4d, black arrows). To determine a possible origin of this epithelial cell debris, we looked for evidence of basal epithelial cell escape from acini at 0.5 months post-wean. We found numerous examples of CK18+ epithelial cells not fully enclosed by the basal myoepithelial cells, as identified by smooth muscle actin positivity (SMA+), as well as examples of epithelial cell protrusion into the stromal compartment. These observations support the idea that during involution, dying epithelial cells may escape the confines of acinar structures and enter lymphatic vessels (Fig. 4d, blue arrows). Altogether, these data support a role for breast lymphatics during the first 0.5 to 1 months post-wean.

COX-2, a rate-limiting enzyme in prostaglandin production and target of NSAIDs, has been implicated in weaning-induced mammary gland lymphangiogenesis in rodents^11^. Further, NSAIDs were found to decrease lymphangiogenesis and reduce metastasis in murine models of PPBC^11,12^. Since previous studies report the mammary epithelial cell as the dominant source of COX-2 in the mammary gland^36^, we evaluated our breast tissue cohort for epithelial cell expression of COX-2. For this analysis, we utilized two COX-2-specific antibody clones reported to differentially recognize posttranslationally modified COX-2, with combined assessment being a better representation of total COX-2 than single antibody staining^37^. Antibody signals were captured by lobule area and grouped by time post-wean, with high and low signals for each antibody clone determined by K-means cluster analysis. Data are displayed as dot plots. This analysis delineates four stain categories as follows: low Ab1/low Ab2; high Ab1/low Ab2; low Ab1/high Ab2; and high Ab1/high Ab2 (Fig. 4e). We find a high number COX-2 positive lobules at 0.5 months post-wean, with 61% of lobules staining positive for either one or both antibody clones. The percent of COX-2+ lobules was highest (93%) at 1 month post-wean before dropping steadily thereafter, reaching 30% positivity by 6–12 months post-wean (Fig. 4e). These COX-2 expression data suggest a potential role for COX-2 in mediating breast lymphangiogenesis that occurs post-wean, and provide further evidence that breast involution in women is most active within the first month post-wean.

## Discussion

Women diagnosed with breast cancer within 10 years of a completed pregnancy have a ~2- to 3-fold increased risk of metastasis compared to age-matched nulliparous women with breast cancer^7,38,39^, even after controlling for tumor size and biologic subtype^2,3^. In rodents, the window of weaning-induced mammary gland involution is a validated hot spot of risk for breast cancer progression^7,11,12,39^, raising the possibility that a similar biology may confer increased risk for breast cancer in young women. Further, in rodent models of PPBC, targeting the active window of involution with short-duration NSAIDs, including ibuprofen, was sufficient to safely mitigate the pro-tumorigenic effects of involution without impacting the normal process of lobule regression and tissue remodeling^12,28,30^. Of potential relevance, the quality of evidence for the safety of ibuprofen in postpartum pain management^40^ and in lactating women and their babies has been determined by The Academy of Breastfeeding Medicine to meet the highest U.S. Preventive Services Task Force level for safety and efficacy^41^. Here, in our healthy young women’s breast study, we sought to characterize the tissue remodeling processes occurring during weaning-induced breast involution and define the duration of this active involution window. We anticipate that understanding weaning-induced breast involution in healthy women will advance a prevention strategy directed against PPBCs.

Using breast biopsies obtained from healthy young women, we find evidence for dramatic lobule expansion and differentiation in the breasts of lactating women compared to nulliparous women, as anticipated. During lactation, we also observed a wide variation in lobular composition between women. Further, we found that classically-defined undifferentiated lobules (types 1 and 2) can express the milk proteins adipophilin and β-casein, albeit at reduced levels compared to large, fully expanded type 4 lobules. These data suggest that during lactation, all lobular subtypes are capable of producing milk, a phenotype previously assigned to type 3 and 4 lobules. With weaning, breast involution is a dominant process that rapidly remodels the lactating breast to a pre-pregnant-like morphology within 2–3 months post-wean. As early as 0.5 months post-wean, we found lactation function declined sharply as demonstrated by loss of milk protein expression and lobules, concurrent with increased epithelial cell death. Further, increased cell death was observed in all lobular subtypes, suggesting that even small lactation-competent lobules might be eliminated with weaning.

In addition to epithelial remodeling, we see evidence of stromal remodeling that includes a CD45+ immune cell infiltrate, lymphangiogenesis, and lymphatic dilation. COX-2, a validated mediator of stromal activation in the involuting rodent mammary gland, peaked in the human breast at 1 month post-wean. These attributes of breast involution are highly reminiscent of the process of weaning-induced mammary gland involution described in rodents^16–18,21,42^. Specifically, previous rodent studies observed increased cell death^14,15,18^, CD45+ inflammation consisting of CD11b+/F480+ MDSCs^27^, M2-skewed macrophages^21,26,43^, and Th-17, Th-2 and Treg-skewed T-cells^21,27,28^, lymphangiogenesis^11,22^, and increased COX-2 expression compared to nulliparous glands^11,12,36^. One limitation of our current study is that the CD45+ immune infiltrate associated with human breast involution was not characterized for specific immune cell populations. Given that immune infiltrates can be pro- or anti-tumor^44–46^, further characterization of the breast immune milieu during weaning-induced involution is warranted.

Another previously described attribute of weaning-induced mammary gland involution is the loosening of mammary epithelial cell tight and e-cadherin+ junctions, where junctional loss is a precursor to programmed cell death of the secretory epithelium^47^. Although our study did not specifically interrogate cellular junctions, we observed basal epithelial cells in direct contact with breast stroma at 0.5 months post-wean, which may indicate compromised epithelial cell junctions. Further, we see increased breast lymphatic density, lymphatic dilation, and epithelial cell debris within the mammary lymphatics—observations that support a role for lymphatics in the clearance of milk components and dying epithelial cells. Of note, previous research has shown that breast cancer cells are resistant to cell death signals activated during involution^20,48^. Thus, loss of epithelial cell junctions may enable anoikis-resistant, early stage breast cancer cells to escape the confines of the ducts and encounter expanded lymphatics, mechanisms anticipated to facilitate systemic dissemination. Whether this route of dissemination partially explains the high rates of metastatic progression observed in young women diagnosed postpartum remains to be determined.

Our finding of natural variation in lobular composition during lactation, with women having breast epithelial tissue ranging from 100% to less than 20% type 4 lobules, raises several questions that may inform weaning-induced involution as a window of breast cancer risk in women. One explanation for variation in lobular composition is that partial gland involution occurs prior to weaning, possibly due to reduced milk demand. If so, we might anticipate lack of lactation proficiency in type 1 and 2 lobules during lactation, as well as a lack of cell death and inflammation in these small lobules upon weaning. In contrast, we find that the type 1 and 2 lobules express the milk proteins adipophilin and β-casein during lactation, suggesting lactation proficiency. Furthermore, we find small lobules undergo cell death and become inflamed upon weaning, data consistent with elimination after weaning. Overall, these data support the hypothesis that lobule involution is a dominant, cell death, and inflammatory biological process that occurs in all lobular subtypes upon weaning.

Others have shown that large, differentiated lobules persist for decades after pregnancy and lactation, and provide compelling data suggesting that such terminal differentiation of lobules confers resistance to subsequent transformational events^31,32^. Although persistence of differentiated lobules was not observed in our study cohort, we cannot rule out the possibility that, in some women, large lobules persist beyond the 2- to 3-month involution window we define here. Based on recently published mouse studies, our data showing heightened immune cell infiltrate and cell death in healthy postweaning breast tissue might also be unanticipated. Specifically, in several mouse models, longer duration of lactation and more gradual weaning are found to protect against breast cancer^49–51^. These gradual weaning studies suggest that rodent models of abrupt weaning may not mirror the breast involution processes in humans, who may be more likely to participate in gradual weaning. In our observational study, the cohort is characterized by long duration of lactation (average of 12 months) and gradual weaning. Yet we observed dominant tissue remodeling during the first months of involution across all lobular subtypes in all cases. Interestingly, in women at 0.5 months post-wean, heterogeneity in lobular composition was not explained by the duration of exclusive nursing of most recent pregnancy, the total duration of lactation, nor the number of childbirths. Combined, these human data further support lobule involution and its associated inflammation as a dominant biological process regardless of breast lobular composition or lactation and weaning patterns.

Our data on normal weaning-induced involution in the human breast may help inform understanding of the dual effect of pregnancy on breast cancer risk, namely the 10- to 20-year period of increased risk followed by reduced risk thereafter^4,38^. One potential misinterpretation of our data is that nursing is a risk factor for breast cancer because it leads to the tumor-promotional attributes of postweaning breast involution. However, numerous studies demonstrate lactation associates with multiple health benefits to mother and child^52–54^, including a long-term protective effect against breast cancer^55–57^. The data from our study are not inconsistent with the protective effect of lactation, but rather suggest that lactation, like pregnancy, may have a dual effect, with lactation conferring long-term protection and postpartum breast involution conferring a transient window of increased risk. We anticipate that the biology of breast involution, not the biology of lactation, drives increased breast cancer risk in postpartum women. In fact, as lobules are laid down during pregnancy, women will experience breast involution regardless of whether they nurse or not. Therefore, avoiding lactation would not be anticipated to mitigate the pro-tumorigenic effects of involution, but may prevent women from benefiting from the protective effect of lactation. Further research is required to better understand the protective effect of lactation and how best to harness it for the prevention of breast cancer.

Our study has several strengths. Postweaning breast tissue samples were obtained from a prospective non-intervention clinical trial in healthy women, with periodic follow-up during lactation in order to identify day of weaning and randomization of women to a single postweaning time point. Additionally, lactation history was collected. All tissue biopsies were taken from the upper-right quadrant of the right breast, to normalize for potential inter-quadrant heterogeneity of tissue composition. Limitations of our study include a lack of ethnic diversity, as the majority of our cohort was non-Hispanic White and reflected the demographics of the study sites. In addition, the biopsies for this study were collected under ultrasound-guided imaging to maximize the amount of fibroglandular tissue obtained. Because of this approach, our tissues are not representative of the relative abundance of fat in the breast and cannot be utilized for adipocyte quantification across a reproductive cycle. We were also unable to determine the source of lobular composition heterogeneity observed in the nulliparous women. Potential factors contributing to lobular composition heterogeneity in nulliparous women include hormonal contraceptives and body mass index; however, what factors regulate lobule differentiation in women remains an open question.

Altogether, our results point to weaning-induced breast involution as a pro-tumorigenic window that can be potentially targeted for the prevention of PPBC. Specifically, many of the mechanisms we identify to be most active in the first months of weaning-induced breast involution, including increased CD45 + inflammation and lymphangiogenesis, are known to be COX-2 dependent in rodents. Importantly, in these preclinical studies, short-term ibuprofen treatment reduced the increased tumor burden and metastasis observed postpartum without impeding clearance of secretory epithelium^28^. Together, the data presented in this study, combined with insights from preclinical research, indicate that COX-2 inhibitors may be safe and effective agents to target the transient window of risk occurring during the first 1–3 months post-wean. Some barriers to the implementation of such a prevention trial include a lack of data as to whether COX-2 inhibition safely reduces the pro-tumor attributes of breast involution in women, and a lack of validated risk assessment tools to identify women at high risk of developing PPBC. However, this study of weaning-induced breast involution in healthy women is a significant step forward for breast cancer prevention in women at risk for PPBC.

## Methods

For this study, we combined breast biopsy samples collected from two prospective biopsy trials of healthy women. The first was our University of Colorado Cancer Center study of women (*n* = 64) undergoing weaning-induced involution and the second was the Komen Benign Tissue Bank at University of Indiana.

The 64 volunteers who provided a single breast biopsy post-wean were randomly assigned to a specific postweaning time point at 0.5, 1, 2, 3, 4–6, or 12 months. Additional tissue from nulliparous, lactation, and time points >12 months post-wean, necessary to complete the reproductive spectrum of our study, were accessed through the Komen Tissue Bank (*n* = 48). As treatment history for Komen cases was available, we confirmed that the Komen cases included in the study were not taking NSAIDs at the time of tissue collection. This combined tissue cohort (*N* = 112) comprised nulliparous (*n* = 17), lactation (*n* = 20), and 0.5 (*n* = 18), 1 (*n* = 17), 2 (*n* = 12), 3 (*n* = 12), 4–6 (*n* = 4), and 12–24 (*n* = 12) months post-wean cases.

For all analyses, tissue was selected for inclusion only if it had adequate epithelial content required for assessment. Epithelial content of biopsies were determined by pathological assessment of H&E-stained sections. For subset analyses, the number of cases included in each subset analysis was determined by power calculations using preliminary data as inputs.

### Ethics

Institutional Review Board (IRB) approval for collection and analysis of human tissues was obtained from the University of Colorado. Tissue provided from the Komen Tissue Bank was analyzed under approval from the Indiana University Institutional Review Board. Informed consent was obtained from all study participants prior to tissue collection using IRB-approved protocols.

### Quantification of lobular composition

Four micrometer formalin-fixed paraffin-embedded (FFPE) H&E-stained sections were utilized to perform lobular analysis on breast biopsies from nulliparous (*n* = 17), lactation (*n* = 20), and 0.5 (*n* = 18), 1 (*n* = 17), 2 (*n* = 12), 3 (*n* = 12), 4–6 (*n* = 4), 12–24 (*n* = 12) months post-wean cases. Analysis was performed on virtual images of slides scanned at 20x magnification (0.4943 μM per pixel) using the Aperio Scanscope AT (Leica Biosystems, Wetzlar, Germany). For each case, lobular composition was determined using pre-defined criteria that account for number of acini per lobule and secretory morphology^11,31^. The lobules were categorized as type 1 (1–15 acini per lobule), 2 (16–50 acini per lobule), or 3 lobules (more than 50 acini per lobule), respectively. Type 4 lobules were defined by distended lumen and flattened acinar epithelium. For each case, every lobule was digitally mapped for lobular subtype, which was used as a reference for subsequent analyses. Lobular composition for each case was defined as an aggregation of all lobule subtypes in that case. All analyses were performed blinded to reproductive group.

### Multiplex Immuno Histochemistry (mIHC) staining, processing, and visualization pipeline

FFPE tissue slides were deparaffinized and pretreated with TRS (Agilent Dako S169984-2) at 125 °C in a pressure cooker (Agilent Dako Cytomation, Inc., Carpenteria, CA, S2800) for 5 min. The tissue was incubated with endogenous block (3% hydrogen peroxide in methanol) for 10 min. Each multiplex immunohistochemistry (mIHC)^28,58^ cycle consisted of protein block, primary and secondary antibody incubations, followed by chromogenic detection. In the beginning of each cycle, the protein block (5% normal goat serum, 2.5% bovine serum albumin, in 1X phosphate-buffered saline) was applied for 10 min followed by a buffer rinse. Next, a single primary antibody, such as adipophilin, β casein, CD45, e-cadherin, SMA, calponin, and D2–40, was applied. Primary antibody details are included in Supplementary Table 1. Depending on the host species of primary antibody generation, anti-rabbit or anti-mouse Simple Stain MAX PO Histofine Peroxidase Polymer (Nichirei Bio chemicals, 414144 or 414134) or anti-rat ImmPRESS Peroxidase Polymer (Vector Laboratories, MP-7444) secondary antibodies were applied next for 30 min, followed by chromogenic detection with peroxidase substrate 3-amino-9-ethylcarbazole (AEC) for signal detection (~20 min). The stained sections were coverslipped and scanned at 20X magnification (0.4943 μM per pixel) using the Aperio Scanscope AT (Leica Biosystems, Wetzlar, Germany). Next, the coverslips were removed gently by immersing slides in buffer solution with agitation, followed by removal of antibody-chromogen complex by microwave stripping in pH 6.0 buffer (Biogenex, #HK086) for 15 min between mIHC cycles. For image processing, the scanned virtual images were aligned in MATLAB version R2019a using the speeded-up robust feature (SURF) algorithm in the Computer Vision Toolbox (The MathWorks, Inc., Natick, MA). Next, images were processed using FIJI (FIJI Is Just ImageJ)^59^. Finally, hematoxylin and AEC signals were extracted from each image using the NIH FIJI plugin RGB_to_CMYK, resulting in AEC signal separation in the yellow channel^60^. For visualization, single-channel images were pseudo-colored and merged in FIJI to generate a multicolor image.

### Quantification of adipophilin expression

To observe the secretory activity of lobules, adipophilin staining was performed on a subset of breast tissue obtained from the nulliparous (*n* = 5), lactation (*n* = 6), 0.5 (*n* = 15), 1 (*n* = 12), 2 (*n* = 9), 3 (*n* = 10), 6–12 (*n* = 3), and 12–24 months post-wean (*n* = 5) cases. A Color Deconvolution algorithm (Aperio Imagescope, Leica, USA) was optimized to capture percent positive tissue area stained for adipophilin within the acinar epithelium of each lobule. Signal from milk in acinar lumen was removed from quantification using negative annotations. The cumulative adipophilin expression for each case was determined and plotted by reproductive group. Using the blueprint for lobular categorization for each case, we assessed the adipophilin expression for each lobular subtype.

### Visualization of dual staining for Adipophilin and β-casein

A subset of cases (lactation = 2, 0.5 months = 4) were stained using the mIHC method for β-casein followed by adipophilin and scanned. The virtual images were processed and visualized as previously described.

### Quantification of immune cell infiltration in lobules

To identify immune cell infiltration, a subset of cases including nulliparous (*n* = 5), lactation (*n* = 5), and 0.5 (*n* = 15), 1 (*n* = 12), 2 (*n* = 9), 3 (*n* = 10), 6–12 (*n* = 3), >12–24 months post-wean (*n* = 5) were stained for CD45 and quantified for percent positive signal in subtype-assigned lobules using an optimized deconvolution algorithm (Aperio Imagescope, Leica, USA). Lobular immune cell infiltrate in each case was plotted by reproductive group and by lobular subtype. Further, to quantify the ratio of intraepithelial to intralobular stromal CD45+ cells in nulliparous (*n* = 5) and 0.5 months post-wean (*n* = 5) cases, we performed manual counts for intraepithelial and intralobular stromal CD45+ cells for five lobules of each lobular subtype, with observer blinded to study group.

### Quantification of epithelial cell death

To quantify cell death during postweaning breast involution, a subset of 0.5 (*n* = 6), 1 (*n* = 6), 2 (*n* = 6), and 3 months (*n* = 6) post-wean were stained for TUNEL+ apoptotic cells and scanned. TUNEL+ cells were counted and normalized to average number of acini per lobule in up to 17 lobules distributed across each case.

### Quantification of lymphatic vessels and lymphatic endothelial cells

To quantify lymphatic vessels, a subset of cases including nulliparous (*n* = 5), lactation (*n* = 6), 0.5 (*n* = 15), 1 (*n* = 12), 2 (*n* = 9), 3 (*n* = 10), and 6–24 months post-wean (*n* = 7) were stained for D2–40. Further, D2–40+ single cells were counted for each case. All counts were assessed by two independent researchers blinded to the reproductive group with discrepancies resolved by consensus. Lymphatic density for each case was assessed by normalizing the lymphatic count to the fibroglandular area of the biopsy. The fibroglandular area was defined as the stromal and lobular area, excluding acinar lumen, adipose tissue, and slide glass. To assess lymphatic function based on whether the majority (>70%) of vessels were collapsed or dilated for each case in nulliparous (*n* = 5), lactation (*n* = 6), and 0.5 (*n* = 6), 1 (*n* = 9), 2 (*n* = 9), 3 (*n* = 10), >12 (*n* = 6) post-wean, a blinded pathological assessment was performed for morphology within 100 μm of lobules. All lymphatics were assessed for dilation, with the cumulative percentage assessed by reproductive group.

### SMA-CK18 dual stain for evidence of basal extrusion

A subset of FFPE breast tissue slides from the 0.5 months post-wean group (*n* = 3) were dual stained for CK18 and SMA for evidence of epithelial cell escape beyond the basal myoepithelial layer (Supplementary Table 1).

### Quantification of COX-2 expression

A subset of cases at 0.5 (*n* = 17), 1 (*n* = 10), 2 (*n* = 9), and 6–12 (*n* = 3) months post-wean were stained for COX-2 expression (Supplementary Table 1). Quantification for percent positive COX-2 signal was performed using a deconvolution algorithm (Aperio Imagescope, Leica, USA). A signal of 0.2% based on K-means clustering was the cutoff for low/high classification for Ab1 (SP21 clone) and a signal of 31.0% was the cutoff for low/high classification for Ab2 (CX229 clone).

### Reporting summary

Further information on experimental design is available in the Nature Research Reporting Summary linked to this paper.

## Supplementary information

Supplementary Documents

Reporting Summary Checklist FLAT

## Data Availability

The data generated and analyzed during this study are described in the following data record: 10.6084/m9.figshare.12869924^61^. The H&E-stained images are available as part of the above data record. In addition, the spreadsheet “Data summary form.xlsx” lists all the .svs files. The spreadsheet “V6 summary sheet_MOM paperchecked2.xlsx” contains the plot data for the figures in the manuscript. Finally, the clinical data regarding lactation history has associated PHI information along with other clinical annotations. The lactation data will be made available upon reasonable request to the corresponding authors.

## Code availability

Quantitative datasets for Figs. 2a, b, 3a, b, and 4a, b, e were generated using the commercially available Aperio Color Deconvolution Algorithm V9 (Leica Biosystems, Wetzlar, Germany) in conjunction with Imagescope V12.4.0.7018 (Leica Biosystems, Wetzlar, Germany). Generation of mIHC images for Figs. 2a, c and 3c were done using a customized pipeline for image alignment, cropping, and color extraction as previously published^28,58^, with access available upon reasonable request.
