# Supplementary material for: Characterization of weaning-induced breast involution in women: implications for young women’s breast cancer
Source: NPJ Breast Cancer. 2020 Oct 16;6:55. doi: 10.1038/s41523-020-00196-3 (PMC7568540; doi:10.1038/s41523-020-00196-3)
Supplement: Supplementary file 1 — Supplementary Documents [file 41523_2020_196_MOESM1_ESM.pdf]

Supplementary Figure 1

A. Lobular composition vs. Length of exclusive nursing

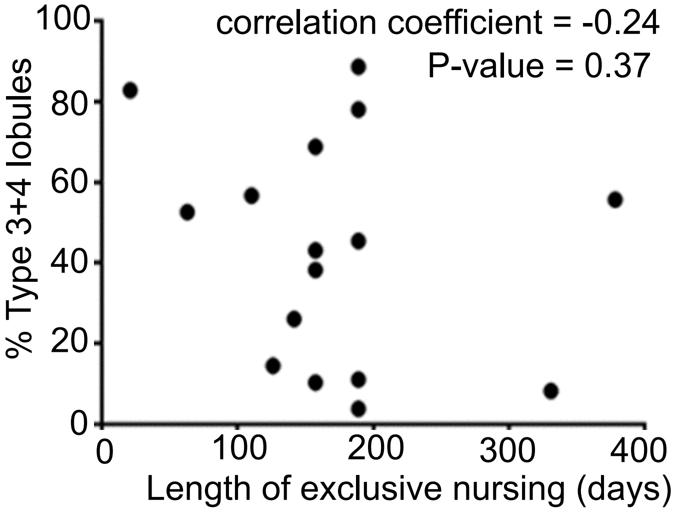

Length of exclusive nursing
Average = 171.6 days (SD ±86.2),
Median = 157.5 days,
Range = 21-378 days

Type 3+4 lobule %
Average = 42.8%,
Median = 44.3%

B. Lobular composition vs. Total lactation time

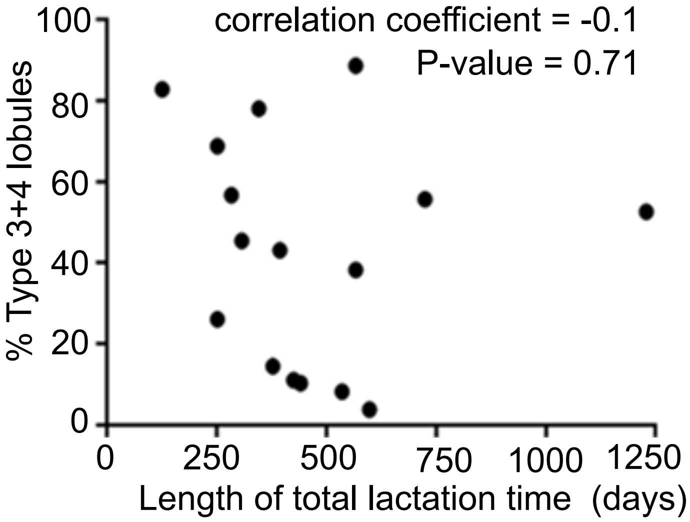

Total lactation time
Average = 464.1 days (SD ±256.4),
Median = 409.5 days,
Range = 126-1228.5 days

Type 3+4 lobule %
Average = 42.8%,
Median = 44.3%

C. Lobular composition vs. Number of births

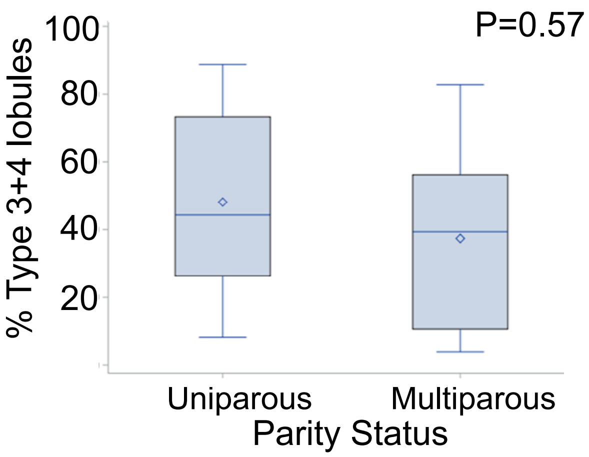

	% Type 3+4 lobules				
Number of Births	Mean	Std. Dev	Min.	Median	Max.
Uniparous	48.1	28.8	8.3	44.3	88.7
Multiparous	37.4	28.5	3.8	39.4	82.8

Supplementary Table 1:

Stain	Antigen	Species	Vendor	Clone	Catalog #	Protein Block (10 min RT)	Concentration	Secondary Antibody	Concentration
Multiplex	Adipophilin	Ms	Lifespan Biosciences	AP125	LS-C348703	5% normal goat serum, 2.5% BSA in 1X PBS	1:300, 1hr RT	Histofine anti Ms (414134F)	ready-to-use, 30 min RT
Multiplex	Beta casein	Ms	Novus Biologicals	F20.14	NB100-2720	5% normal goat serum, 2.5% BSA in 1X PBS	1:200, 1hr RT	Histofine anti Ms (414134F)	ready-to-use, 30 min RT
Multiplex	CD45 (Leukocyte Common Antigen)	Ms	Dako	2B11 + PD7/26	M0701	5% normal goat serum, 2.5% BSA in 1X PBS	1:200, 1hr RT	Histofine anti Ms (414134F)	ready-to-use, 30 min RT
Multiplex	Podoplanin	Ms	Dako	D2-40	M3619	5% normal goat serum, 2.5% BSA in 1X PBS	1:50, 1hr RT	Histofine anti Ms (414134F)	ready-to-use, 30 min RT
Multiplex	E-cadherin	Rb	Cell Signaling Technology	24E10	3195	5% normal goat serum, 2.5% BSA in 1X PBS	1:100, 1hr RT	Histofine anti Rb (414144F)	ready-to-use, 30 min RT
Dual	SMA	Ms	Dako	1A4	M0851	Biocare Background Sniper #CB917	1:1000, 1hr RT	DAKO Envision+HRP anti Ms (K4001)	ready-to-use, 30 min RT
Dual	Cytokeratin 18 (CK18)	Rb	abcam	EPR17347	ab181597	Biocare Background Sniper #CB917	1:5000, 1hr RT	PolyviewPlus AP Polymer anti Rb (ENZ-ACC110-0150)	ready-to-use, 30 min RT
Dual	COX-2	Rb	Thermo Scientific	SP21	RM-9121	Biocare Background Sniper #CB917	1:150, 1hr RT	PolyviewPlus AP Polymer anti Rb (ENZ-ACC110-0150)	ready-to-use, 30 min RT
Dual	COX-2	Ms	Cayman Chemical	CX229	160112	Biocare Background Sniper #CB917	1:250, 1.5 hr RT	DAKO Envision+HRP anti Ms (K4001)	ready-to-use, 30 min RT
