# Supplementary material for: Characterization of weaning-induced breast involution in women: implications for young women’s breast cancer
Source: NPJ Breast Cancer. 2020 Oct 16;6:55. doi: 10.1038/s41523-020-00196-3 (PMC7568540; doi:10.1038/s41523-020-00196-3)
Supplement: Supplementary file 2 — Reporting Summary Checklist FLAT [file 41523_2020_196_MOESM2_ESM.pdf]

nature research

Corresponding author(s): Pepper Schedin, NPJBCANCER-00643

Last updated by author(s): Jul 22, 2020

Reporting Summary

Nature Research wishes to improve the reproducibility of the work that we publish. This form provides structure for consistency and transparency in reporting. For further information on Nature Research policies, see Authors & Referees and the Editorial Policy Checklist.

Statistics

For all statistical analyses, confirm that the following items are present in the figure legend, table legend, main text, or Methods section.

n/a	Confirmed	
☐	☒	The exact sample size (*n*) for each experimental group/condition, given as a discrete number and unit of measurement
☐	☒	A statement on whether measurements were taken from distinct samples or whether the same sample was measured repeatedly
☐	☒	The statistical test(s) used AND whether they are one- or two-sided<br>*Only common tests should be described solely by name; describe more complex techniques in the Methods section.*
☐	☒	A description of all covariates tested
☒	☐	A description of any assumptions or corrections, such as tests of normality and adjustment for multiple comparisons
☐	☒	A full description of the statistical parameters including central tendency (e.g. means) or other basic estimates (e.g. regression coefficient) AND variation (e.g. standard deviation) or associated estimates of uncertainty (e.g. confidence intervals)
☒	☐	For null hypothesis testing, the test statistic (e.g. *F*, *t*, *r*) with confidence intervals, effect sizes, degrees of freedom and *P* value noted<br>*Give P values as exact values whenever suitable.*
☒	☐	For Bayesian analysis, information on the choice of priors and Markov chain Monte Carlo settings
☒	☐	For hierarchical and complex designs, identification of the appropriate level for tests and full reporting of outcomes
☒	☐	Estimates of effect sizes (e.g. Cohen's *d*, Pearson's *r*), indicating how they were calculated

Our web collection on statistics for biologists contains articles on many of the points above.

Software and code

Policy information about availability of computer code

Data collection	Commercial Aperio ImageScope analysis software V9(Leica Biosystems, Vista, CA) was used in data collection
Data analysis	Commercial Graph Pad prism version 8.0 was used for the statistical analysis of data in the manuscript

For manuscripts utilizing custom algorithms or software that are central to the research but not yet described in published literature, software must be made available to editors/reviewers. We strongly encourage code deposition in a community repository (e.g. GitHub). See the Nature Research guidelines for submitting code & software for further information.

Data

Policy information about availability of data

All manuscripts must include a data availability statement. This statement should provide the following information, where applicable:

- Accession codes, unique identifiers, or web links for publicly available datasets
- A list of figures that have associated raw data
- A description of any restrictions on data availability

Provide your data availability statement here.

Field-specific reporting

Please select the one below that is the best fit for your research. If you are not sure, read the appropriate sections before making your selection.

☒ Life sciences ☐ Behavioural & social sciences ☐ Ecological, evolutionary & environmental sciences

For a reference copy of the document with all sections, see nature.com/documents/nr-reporting-summary-flat.pdf

Life sciences study design

All studies must disclose on these points even when the disclosure is negative.

Sample size	De-identified FFPE breast biopsy samples collected from two prospective biopsy trials of healthy women were used in the study. The first was our University of Colorado Cancer Center (UCCC) study of women (n = 64) undergoing weaning-induced involution and the second was the Komen Benign Tissue Bank at University of Indiana. In the UCCC cohort healthy women provided a single breast biopsy post-wean. Each women was randomly assigned to a specific post-wean time point at 0.5, 1, 2, 3, 4-6 or 12 months. Additional tissue from nulliparous, lactation, and time points >12 months post-wean, necessary to complete the reproductive spectrum of our study were accessed through the Komen Tissue Bank (n = 48). This combined tissue cohort (N = 112) is comprised of nulliparous (n = 17), lactation (n = 20), and 0.5 (n = 18), 1 (n = 17), 2 (n = 12), 3 (n = 12), 4-6 (n = 4), and 12-24 (n = 12) months post-wean cases.
Data exclusions	For all analyses, tissue was selected for inclusion only if it had adequate epithelial content required for assessment based on H&E staining.
Replication	All data analysis was performed by a researcher and cross checked by another researcher on the team. Any data point discrepancy was discussed and resolved to a consensus agreement.
Randomization	In the UCCC cohort healthy women provided a single breast biopsy post-wean. Each women was randomly assigned to a specific post-wean time point at 0.5, 1, 2, 3, 4-6 or 12 months. For subset analyses, number of cases included in each subset analyses was determined by power calculations using preliminary data as inputs.
Blinding	All data acquisition was performed by investigators who were blinded to study group

Reporting for specific materials, systems and methods

We require information from authors about some types of materials, experimental systems and methods used in many studies. Here, indicate whether each material, system or method listed is relevant to your study. If you are not sure if a list item applies to your research, read the appropriate section before selecting a response.

Materials & experimental systems

n/a	Involved in the study	
☐	☒	Antibodies
☒	☐	Eukaryotic cell lines
☒	☐	Palaeontology
☒	☐	Animals and other organisms
☐	☒	Human research participants
☒	☐	Clinical data

Methods

n/a	Involved in the study	
☒	☐	ChIP-seq
☒	☐	Flow cytometry
☒	☐	MRI-based neuroimaging

Antibodies

Antibodies used	Primary antibodies used included Adipophilin (Lifespan Biosciences, #LS-C348703,clone AP125), Beta casein (Novus Biologicals, #NB100-2720, clone F20.14), CD45 (Dako, #M0701, clone2B11 + PD7/26), Podoplanin (Dako, #M3619, clone D2-40), E-cadherin (Cell Signaling Technology, #3195, clone 2.40E+11), SMA (Dako, #M0851, clone 1A4), Cytokeratin 18 (Abcam, #ab181597, clone EPR17347), Cox-2 (Thermo Scientific, #RM-9121, clone SP21 and Cayman Chemical, #160112, clone CX229), secondary anti-rabbit or anti-mouse Simple Stain MAX PO Histofine Peroxidase Polymer (Nichirei Biochemicals, #414144 or #414134, RTU) or anti-rat ImmPRESS Peroxidase Polymer (Vector Laboratories, #MP-7444,RTU)
Validation	All Cell Signaling Technology antibodies are certified as meeting the quality control standards of Cell Signaling Technology per certificates of analysis using authentication methods such as Western blot analyses with siRNA knockdown, use of positive and negative tissue, cell extracts, and xenografts with known target expression, and use of blocking peptides where possible. For all abcam products, application notes include validated applications per Western blotting and tissue microarray staining, and recommended starting dilutions, with optimal conditions determined by end-user. All Dako antibodies are validated for In vitro diagnostic use and are authenticated for western blots, positive and negative tissue controls.

Human research participants

Policy information about studies involving human research participants

Population characteristics	The study includes Formalin-fixed paraffin-embedded (FFPE) human breast tissue samples from healthy women aged >20 years who donated a one time breast biopsy .
Recruitment	Participants were recruited to the UCCC study. This study was prospectively conducted using the Formalin-fixed paraffin-embedded (FFPE) human breast tissue samples collected by UCCC and Komen tissue bank.

Ethics oversight

Formalin-fixed paraffin-embedded (FFPE) human breast tissue for this study was approved by Komen tissue bank repository and Institutional Review Boards at Colorado Multiple Institution Review Board (COMIRB), and Oregon Health and Science University (OHSU).

Note that full information on the approval of the study protocol must also be provided in the manuscript.
